# The norpurpureine alkaloid from *Annona purpurea* inhibits human platelet activation in vitro

**DOI:** 10.1186/s11658-018-0082-4

**Published:** 2018-04-18

**Authors:** Gabriela Sánchez, Omar Estrada, Giovana Acha, Alfonso Cardozo, Franshelle Peña, Marie Christine Ruiz, Fabián Michelangeli, Claudia Alvarado-Castillo

**Affiliations:** 1 Centro de Biofísica y Bioquímica (CBB), Instituto Venezolano de Investigaciones Científicas (IVIC), Caracas, Bolivarian Republic of Venezuela; 2 Laboratorio de Botánica Sistemática, Facultad de Agronomía, Universidad Central de Venezuela (UCV), Maracay, Bolivarian Republic of Venezuela; 3 Laboratorio de Hemostasia y Genética Vascular, Centro de Biofísica y Bioquímica, Instituto Venezolano de Investigaciones Científicas, Apartado 20632, K11 de la Carretera Panamericana, Caracas, 1020-A Bolivarian Republic of Venezuela

**Keywords:** *Annona purpurea*, Alkaloids, Norpurpureine, Human platelets

## Abstract

**Background:**

The leaves of *Annona purpurea* have yielded several alkaloids with anti-aggregation activities against rabbit platelets. This is promising in the search for agents that might act against platelets and reduce the incidence of cardiovascular diseases. Since significant differences in platelet function have been reported between human and animal platelets, a study focusing on the effect of *A. purpurea* extracts against human platelet activation is necessary.

**Methods:**

The compounds in an *A. purpurea* ethanolic extract underwent bio-guided fractionation and were used for in vitro human platelet aggregation assays to isolate the compounds with anti-platelet activity. The bioactive compounds were identified by spectroscopic analysis. Additional platelet studies were performed to characterize their action as inhibitors of human platelet activation.

**Results:**

The benzylisoquinoline alkaloid norpurpureine was identified as the major anti-platelet compound. The IC_50_ for norpurpureine was 80 μM against platelets when stimulated with adenosine 5′-diphosphate (ADP), collagen and thrombin. It was pharmacologically effective from 20 to 220 μM. Norpurpureine (220 μM) exhibited its in vitro effectiveness in samples from 30 healthy human donors who did not take any drugs during the 2 weeks prior to the collection. Norpurpureine also gradually inhibited granule secretion and adhesion of activated platelets to immobilized fibrinogen. At the intra-platelet level, norpurpureine prevented agonist-stimulated calcium mobilization and cAMP reduction. Structure–activity relationship analysis indicates that the lack of a methyl group at the nitrogen seems to be key in the ability of the compound to interact with its molecular target.

**Conclusion:**

Norpurpureine displays a promising in vitro pharmacological profile as an inhibitor of human platelet activation. Its molecular target could be a common effector between Ca^2+^ and cAMP signaling, such as the PLC-PKC-Ca^2+^ pathway and PDEs. This needs further evaluation at the protein isoform level.

**Electronic supplementary material:**

The online version of this article (10.1186/s11658-018-0082-4) contains supplementary material, which is available to authorized users.

## Background

Platelet activation is a key event in thrombus formation, chronic inflammation and atherosclerosis, which are all multicellular processes involved in the development of cardiovascular diseases. Currently, anti-platelet agents such as aspirin and clopidogrel are widely used alone or in combination to reduce the incidence of ischemic stroke and to prevent arterial thrombosis [1]. Despite the efficacy of dual anti-aggregation therapy, growing evidence of drug resistance for aspirin and clopidogrel [2] highlight the need to search for novel anti-platelet agents to reduce the incidence of cardiovascular diseases; which are the globally leading causes of death and disability [3].

Various compounds with platelet anti-aggregation activity have been isolated from medicinal plants using animal platelets and in vitro bio-assays [4]. Several anti-platelet compounds have been identified from different species of the *Annona* genus (Annonaceae) using rabbit platelets: acid amines from *A. montana* [5], aporphine alkaloids from *A. purpurea* [6, 7] and ent-kaurane diterpenoids from *A. squamosa* [8]. *A. purpurea* is widely distributed throughout the tropical and subtropical regions of Central America [9], making it an attractive source for pharmacologically active substances.

Significant differences in platelet function may exist between human and animal platelets [10–13], so these effects on rabbit platelets need to be assessed using a human model. In this study, we searched for anti-platelet compounds in the leaves of *A. purpurea*, using human platelets and a bio-guided fractionation of its ethanolic extract (EE). Additionally, we explored the pharmacological properties and mechanism of action of the isolated bioactive compounds.

## Methods

### Materials

Acetone, acetonitrile and acetic acid (reagent grade) were purchase from J.T. Baker Chemical. Methanol, ethanol, DMSO, ADP, ATP, thrombin, human fibrinogen, IBMX, PMA, probenecid, apyrase, cAMP enzyme immunoassay kit, Sephadex LH-20 and dimethylsulfoxide-d6 to NMR were from Sigma-Aldrich. Collagen was from Helena Laboratories. CHRONO-LUME was from Chrono-log Corporation and Fura-2-AM was from Invitrogen Corporation.

### Plant material

The leaves of *Annona purpurea* Moc. & Sessé ex Dunal [14] were collected in July 2008 at Parque Nacional Henri Pittier, Aragua, Venezuela. They were identified by Dr. Alfonzo Cardozo and a voucher specimen (AC27435) was deposited in the herbarium of Víctor Manuel Badillo (MY), Facultad de Agronomía, UCV, Maracay, Venezuela. The collection of plant material was done in accordance with the Biological Diversity Law of República Bolivariana de Venezuela (gaceta oficial número 5.468 extraordinario de fecha 24–05-2000), under permission number H-46 (date of issue: August 1, 2007; date of expiration: August 1, 2008). This was granted to the herbarium of Victor Manuel Badillo (MY) in the name of Alfonzo José Cardozo López and allows the collection of botanical samples for scientific research purposes.

### Blood collection and platelet preparations

Blood was obtained by clean venipuncture from 30 healthy human donors who did not take any drugs during the 2 weeks prior to the collection. All donors gave informed consent for the study, which was approved by the Bioethical Committee of IVIC (number 1316, approval on March 2009), following the guidelines of the Declaration of Helsinki and Tokyo for humans.

Platelet-rich plasma (PRP) and washed platelets (WP) were obtained according to the method of Cazenave et al. [15], with modifications. Briefly, blood samples were collected, discarding the first 2 or 3 ml, into 3.2% (109 mM) trisodium citrate didydrate (1:9 *v*/v, citrate to blood) and centrifuged at 160×g for 15 min (with no brake), at room temperature (RT). PRP (the upper phase) was isolated and the remaining lower phase was further centrifuged at 1500×g for 15 min at RT to obtain platelet-poor plasma (PPP), which is used to determine 100% light transmittance in platelet aggregation assays. WP were prepared from the PRP using blood anticoagulated with acid–citrate–dextrose (ACD) consisting of 38 mM citric acid monohydrate, 85 mM trisodium citrate dihydrate, and 123 mM anhydrous D(+)glucose at pH 5 (1:6 v/v, ACD to blood). Then, PRP supplemented with 1 μM PGE_1_ was centrifuged at 1500×g for 15 min at RT and the platelet pellet was washed once with HEPES-modified Tyrode buffer consisting of 134 mM NaCl, 2.9 mM KCl, 12 mM NaHCO_3_, 0.34 mM NaH_2_PO_4_, 1 mM MgCl_2_, 20 mM HEPES (pH 6.5) and 5 mM glucose; supplemented with 1 μM PGE_1_. The washed platelet pellet was carefully and slowly resuspended at 300,000 platelets/μl in HEPES-modified Tyrode’s buffer pH 7.4; supplemented with 0.35% albumin, 0.5 U/ml apyrase and 2 mM CaCl_2_, without PGE_1_. The resuspended platelets were rested at RT for at least 30 min and 0.25 mg/ml human fibrinogen was added prior to use.

### In vitro platelet aggregation assay

Platelet aggregation was monitored using Born’s turbidimetric method [16]. Inhibition experiments were done as described earlier [17] by incubating the platelets with different *A. purpurea* samples, norpurpureine and purpureine for 10 min before stimulation with the agonists: 10 μM ADP, 1 μg/ml collagen (in PRP) and 0.075 U/ml thrombin (in WP). DMSO was used as a vehicle at a final concentration less than 0.25% in all the cases. Platelet aggregation responses were recorded for 10 min (Chrono-log 700), at 37 °C with stirring at 1000 rpm.

### Bioassay-guided isolation and identification of anti-platelet compounds from *A. purpurea* leaves

The anti-aggregation activities of the *A. purpurea* extract, fractions and isolated compounds against human platelets in vitro were tested at 250 μg/ml. Only the active samples were then studied in the next phase of the separation process.

Powdered dried leaves (300 g) were subjected to percolation with ethanol for a week. The solvent was evaporated in vacuo to yield 52 g of dry ethanol extract (EE). Then, from the partition of *A. purpurea* EE in methanol–water (1:1) two fractions were obtained: a green residue (21.2 g) named methanol–water insoluble fraction (MWIF); and a red solution that after evaporation in vacuo yielded a red residue (27.7 g) named methanol–water soluble fraction (MWSF). MWSF was repeatedly extracted with acetone to obtain two new fractions: a brownish residue named acetone insoluble fraction (AIF); and a yellowish solution from which a yellowish residue named AF was yielded (11.7 g) after evaporation of the solvent. A portion of AF (2 g) was fractionated on Sephadex LH-20 column chromatography (CC) using methanol as the eluent to give three fractions, named I–III. From fraction II, two compounds: norpurpureine (**A**; 350 mg) and purpureine (**B**; 200 mg) were finally purified after CC on RP-18 using the mixture acetonitrile–water–acetic acid (65–30–0.5) as the eluent. The structures of compounds **A** and **B** were characterized in 1D and 2D NMR experiments and identified by comparison with spectroscopic data [18].

### Spectroscopic analysis

^1^H vand ^13^C NMR spectra were performed in hexadeuterodimethylsulphoxide (DMSOd6) on a Brucker DRX 500 spectrometer at Centro de Química, IVIC. Mass spectra were measured in a Bruker Micro TOF-QIII spectrometer set to ESI mode using MeOH as the solvent at Centro de Biología Estructural, IVIC. NMR spectra of norpurpureine and purpureine (Additional file 1) and MS spectra of norpurpureine and purpureine (Additional file 2), are available.

### Measurements of ATP secreted from activated platelets

The ATP released from platelets (0.4 ml PRP adjusted to 3.0 × 10^8^/ml) was measured by adding 50 μl of luciferin/luciferase reagent (CHRONO-LUME), 1 min before stimulation with 10 μM ADP. Platelet aggregation and ATP secretion responses were simultaneously measured, at 37 °C with stirring at 1000 rpm, in a Lumi-aggregometer Model 700 (Chrono-Log Co.). The amount of ATP (nmols) was determined using ATP standard calibration.

### Adhesion assay of activated human platelets onto fixed fibrinogen

Platelets (1 × 10^7^) in PRP pre-treated with DMSO, apyrase or norpurpureine for 10 min (at the indicated concentrations) were stimulated with 10 μM ADP for 3 min and then platelet adhesion onto fibrinogen-coated wells was allowed for 1 h at RT. The measurements of adherent platelets to fibrinogen were carried out according to Eriksson and Whiss [19].

### Quantification of cytosolic calcium concentrations

Platelets in PRP supplemented with 2 mM probenecid were incubated with the fluorescent calcium indicator Fura-2-AM (5 μM) at 37 °C for 1 h in darkness. The washed pellet of Fura-2-AM-loaded platelets were resuspended with HEPES buffer supplemented with 2 mM probenecid and 2 mM CaCl_2_, at a concentration of 1 × 10^9^ platelets/ml. Platelets (1 ml) were incubated with DMSO or norpurpureine for 10 min before addition of 0.075 U/ml thrombin. The measurements of [Ca^2+^]_i_ were performed at 37 °C in a MSIII fluorometer (Photon Technology International) equipped with a stirrer and temperature control, using alternate excitation wavelengths of 340 and 380 nm and an emission wavelength of 510 nm as previously described [20]. The [Ca^2+^]_i_ values were calculated using the SPEX dM3000 software package, according to the equation described by Grynkiewicz et al. [21].

### Measurements of cAMP levels in platelets

Intra-platelet cAMP concentrations were measured using a commercially available kit. Briefly, platelets (3 × 10^8^/ml) were pre-incubated as indicated, for 10 min at 37 °C with stirring at 1000 rpm, before agonist stimulation with 10 μM ADP, 1 μg/ml collagen (in PRP) and 0.075 U/ml thrombin (in WP). The reaction was stopped by the addition of an equal volume of ice-cold ethanol; the samples were kept on ice for a further 45 min and centrifuged at 7000×g for 15 min at 4 °C. Each supernatant containing cAMP was evaporated to dryness in vacuo and reconstituted with water. cAMP levels were determined according to the manufacturer’s specifications (cAMP Enzyme Immunoassay Kit, Sigma-Aldrich).

### Statistical analysis

Values are expressed as means and standard deviations (SD). Statistical analysis was performed applying one-way ANOVA and Bonferroni post hot test using GraphPad Prism 6.1 software. Differences between responses were considered statistically significant at *p* < 0.05(*), 0.01(**) and 0.001(***).

## Results

### Norpurpureine, the major anti-platelet compound isolated from *A. purpurea* leaves

**Fig. 1:**
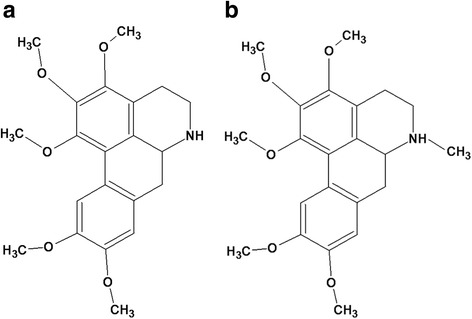
The structures of norpurpureine (**a**) and purpureine (**b**) isolated from *A. purpurea* leaves

We found that *A. purpurea* EE at 250 μg/ml exhibited modest anti-platelet aggregation effects against ADP, collagen and thrombin (between 30 and 45%, *n* = 5 per agonist). Bio-directed fractionation of this EE led to the isolation and identification of two known alkaloids: norpurpureine and purpureine (Fig. 1).

**Fig. 2:**
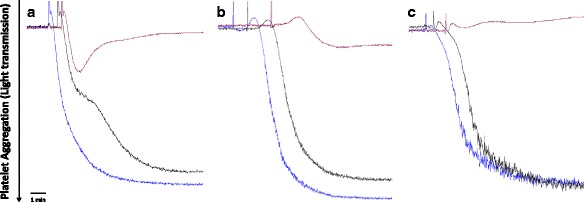
Norpurpureine is a non-selective inhibitor of human platelet aggregation. Typical traces of platelet aggregation responses monitored by changes in the light transmission signal over time are shown. Platelets were incubated with the vehicle (0.25% DMSO, blue), 250 μM purpureine (black) or 250 μM norpurpureine (red) for 10 min before their stimulation with 10 μM ADP (**a**), 1 μg/ml collagen (**b**) (in PRP) or 0.075 U/ml thrombin (**c**) (in WP). These original tracings are representative of five curves done for each agonist

Interestingly, norpurpureine at 250 μM (*n* = 5 per agonist) completely retained the anti-platelet effects observed for *A. purpurea* EE, while purpureine at 250 μM (*n* = 5 per agonist) did not inhibit the aggregation of human platelets (Fig. 2). These findings indicate that norpurpureine is the major anti-platelet compound in *A. purpurea* leaves. Importantly, norpurpureine (100 μg/ml for 30 min, without agonists) alters neither the number and morphology of platelets (40×, under light microscopy) nor the approximate number of platelet microparticles (mostly visible like bacteria at 100×). This reveals that this alkaloid did not induce signs of platelet activation or toxicity by itself under the experimental conditions.

### Efficacy, potency and effectiveness of norpurpureine as inhibitor of human platelet activation

**Fig. 3:**
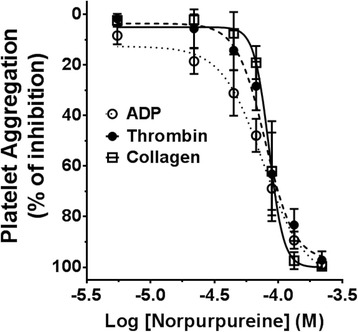
Potency of norpurpureine as an anti-platelet agent. Concentration–response curves for norpurpureine inhibitory actions on agonist-induced platelet aggregations were constructed. Each data point, mean ± SD (*n* = 5), is presented as the percentage reduction of the maximal amplitude response (versus the control, in the absence of norpurpureine). All measures were taken 10 min after the addition of the stimuli

Norpurpureine inhibited agonist-induced platelet aggregation in a concentration-dependent manner, showing a pharmacologically effective concentration range of 20 μM [10^(− 4.70)] to 220 μM [10^(− 3.65)], for each agonist: ADP, collagen and thrombin (Fig. 3). The Hill slopes of these three curves (2.7 ± 0.8 for ADP; 7.53 ± 2.9 for collagen; and 3.9 ± 1.9 for thrombin; *p* = 0.056) and the concentration values at which norpurpureine inhibits 50% of the maximal response (IC_50_) (ADP at 77.6 ± 8 μM; collagen at 84.5 ± 4 μM; and thrombin at 79.4 ± 9 μM; *p* = 0.44) are similar, indicating potency as anti-platelet agent around 80 μM (29.71 μg/ml). It is remarkable that norpurpureine was pharmacologically effective (at 220 μM) in samples from 30 apparently healthy blood donors, providing evidence of its in vitro effectiveness as an anti-platelet agent.

### Norpurpureine inhibits platelet secretion

**Fig. 4:**
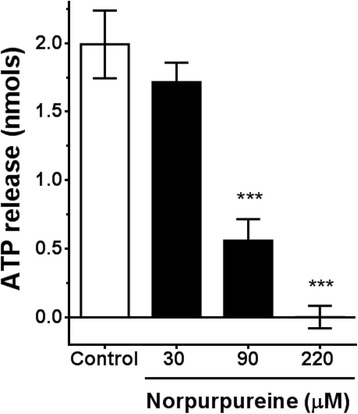
Norpurpureine inhibits platelet secretion. Platelets were stimulated with 10 μM ADP in the absence or presence of the indicated concentrations of norpurpureine. The amount of ATP released was quantified from the bioluminescence of the ATP–luciferin/luciferase reaction. The ATP standard calibration curve was used for quantification of the nucleotide. The data are the means ± SD (*n* = 3, done in triplicate), *p* < 0.001 (***) vs. vehicle

Since granule secretion is a common amplification event during platelet activation, we evaluated the ability of norpurpureine to modulate the amount of ATP released from dense granules of platelets activated by ADP (Fig. 4). Norpurpureine inhibited the agonist-induced secretory response in a concentration-dependent manner, exhibiting a significant inhibitory response near its IC_50_ value (80 μM).

### Norpurpureine prevents the adhesion of activated platelets to fibrinogen

As the formation of fibrinogen bridges between adjacent activated platelets is an important step for platelet aggregation, we evaluated whether norpurpureine affected the binding of fibrinogen to its receptor (activated integrin αIIbβ3) during platelet activation. In Table 1, norpurpureine gradually prevents the adhesion of ADP-stimulated platelets onto fixed fibrinogen. This is similar to the effect of apyrase, a phosphatase that avoids platelet activation by dephosphorylation of ADP to AMP. These results suggest that norpurpureine inhibits the agonist-induced activation (inside-out) of the integrin αIIbβ3, thereby preventing fibrinogen binding to activated platelets.

**Table 1:** Norpurpureine prevents ADP-induced platelet adhesion onto fixed fibrinogen

	Net Adhesion (AU)		Platelet-specific adhesion (PSA) to Fg induced by ADP	PSA (%) vs. vehicle
	ADP (−)	ADP (+)		
Vehicle	0.39 (0.04)	0.95 (0.05)	0.56 (0.05)	100.00 (4.7)
Norp 220 μM	0.42 (0.03)	0.51 (0.04)	0.09 (0.01)	16.07 (1.8)^***^
Norp 90 μM	0.41 (0.02)	0.73 (0.04)	0.32 (0.02)	56.55 (3.7)^***^
Norp 30 μM	0.42 (0.03)	0.85 (0.02)	0.43 (0.03)	76.78 (4.7)^**^
Apyrase	0.40 (0.02)	0.49 (0.03)	0.09 (0.02)	16.67 (2.7)^***^

Platelets in PRP (1 × 10^5^) were pretreated with vehicle (DMSO 0.25%), apyrase (0.5 U/ml) or norpurpureine (Norp) at the indicated concentrations for 10 min before the addition of 10 μM ADP to test their adhesion onto fixed fibrinogen (Fg). The values are the means ± SD (*n* = 3, done in triplicate) *p* < 0.01 (**) and *p* < 0.001 (***) compare to vehicle.

### Norpurpureine blocks the agonist-induced elevation of [Ca^2+^]_i_ in human platelets

**Fig. 5:**
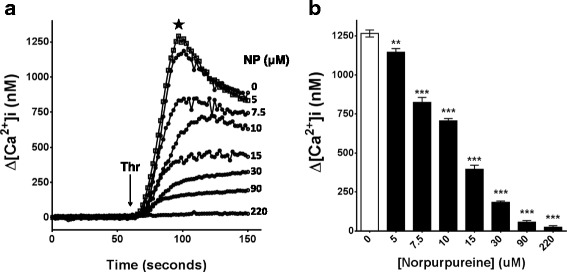
Norpurpureine inhibits agonist-induced elevation of [Ca^2+^]_i_ in human platelets. **a** Typical superimposed traces of [Ca^2+^]_i_ changes stimulated by 0.075 U/ml thrombin (Thr) over time, in Fura-2-AM-loaded platelets pretreated with norpurpureine at the indicated concentrations. **b** Concentration-dependent inhibition of norpurpureine on the maximum elevation (★ in 5A, for each curve) reached by [Ca^2+^]_i_ after thrombin stimulation. Each data point is the mean ± SD (*n* = 3), *p* < 0.01(**), *p* < 0.001(***) vs. vehicle

Given the critical role of [Ca^2+^]_i_ elevation during platelet activation [22], we investigated whether norpurpureine could modulate the [Ca^2+^]_i_ changes induced by thrombin on human platelets (Fig. 5). Norpurpureine prevents the agonist-stimulated elevation of [Ca^2+^]_i_, reducing the amplitude of the response in a concentration-dependent manner (Fig. 5a). This inhibitory effect became significant from a low micromolar range ~ 5 μM (Fig. 5b) and was almost completely blocked around 80 μM (IC_50_).

### Protein kinase C activation reversed the inhibitory action of norpurpureine on agonist-stimulated platelet aggregation

**Fig. 6:**
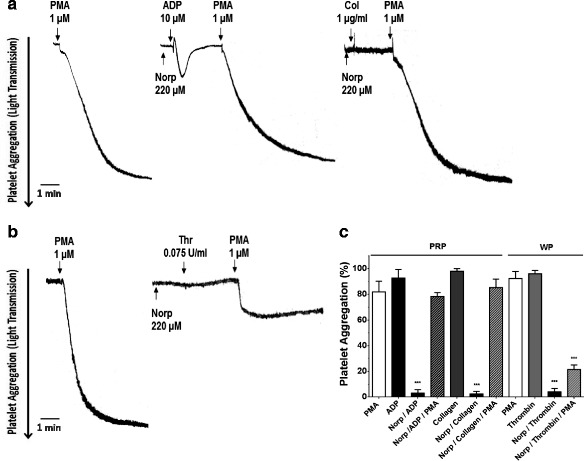
PKC activation reversed the inhibition of norpurpureine on agonist-stimulated platelet aggregation. Typical traces of platelet aggregation monitored by changes in the light transmission signal over time are shown in **a** platelet-rich plasma (PRP) and **b** washed platelets (WP). Platelets were exposed to norpurpureine inhibitory action for 10 min, then challenged with ADP and collagen (Col) or thrombin (Thr) followed by PMA, as indicated. PMA (control) in each platelet preparation is shown. These original tracings are representative of three experiments done independently for each agonist. Quantification of the data in (**a**) and (**b**) are shown in (**c**). Each data point is the mean ± SD (*n* = 3), *p* < 0.001(***) vs. PMA in each platelet preparation

Protein kinase C (PKC) is a common effector of signaling pathways triggered by the activation of different platelet receptors [23]. Therefore, we tested whether the anti-platelet effect of norpurpureine was affected by PKC activation (Fig. 6). We found that the phorbol ester PMA, a non-selective PKC activator, totally reversed the inhibition of norpurpureine on platelet aggregations stimulated by ADP and collagen but only partially reversed that inhibitory response on platelets stimulated by thrombin.

These results suggest that norpurpureine is an inhibitor of PKC activation or that its molecular target is upstream of PKC. This also reveals a complex regulation of PKC in human platelets, supporting evidence that platelet secretion and integrin activation could be positively or negatively regulated by different isoforms of PKC, which in turn may differ from one agonist to another [23].

### Norpurpureine prevents the agonist-induced decrease in cAMP levels in human platelets

**Fig. 7:**
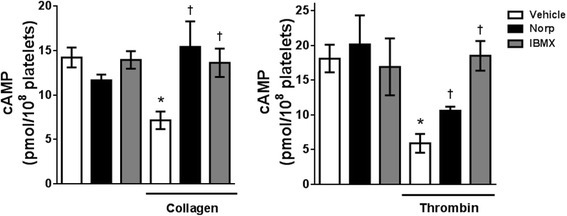
Norpurpureine prevents the agonist-induced decrease in intra-platelet cAMP levels. Platelets were pre-treated for 10 min with vehicle (0.25% DMSO), 220 μM norpurpureine and 10 μM IBMX; and stimulated with 1 μg/ml collagen (in platelet-rich plasma, PRP) and 0.075 U/ml thrombin (in washed platelets, WP). Data are the means ± SD (*n* = 2, done in triplicate). *p* < 0.05 (*) compare to basal (PRP or WP) and *p* < 0.05 (†) compared to collagen- or thrombin-activated platelets

Activation of platelets involves the reduction of intracellular cAMP levels, mainly due to inhibition of adenylyl cyclases or activation of phosphodiesterases (PDEs), which are respectively enzymes that catalyze its synthesis and degradation [24]. Therefore, we investigated the effect of norpurpureine on the changes in intra-platelet cAMP levels after agonist-induced platelet activation. As shown in Fig. 7, norpurpureine and IBMX, a non-selected inhibitor of PDEs, did not modify the cAMP levels of resting platelets but significantly prevented the reductions in basal cAMP levels induced by collagen and thrombin. Similar results were obtained for ADP. Thus, the inhibitory action of norpurpureine involves prevention of cAMP degradation and it may be acting as an inhibitor of platelet PDEs.

## Discussion

In this study, we used *A. purpurea* leaves collected in Maracay, Venezuela and human platelets. We found that the *A. purpurea* EE inhibited the aggregation of human platelets induced by ADP, collagen and thrombin; and that these anti-aggregating activities were retained by the alkaloid fraction. Similar results were reported by Chang et al. [6, 7] for *A. purpurea* extract (leaves collected in Chia-Yi, Taiwan) using rabbit platelets. Those authors identified nine [6] and five [7] alkaloids with anti-platelet actions.

In this work, we identified two of the five known alkaloids isolated by Chang et al. [7]: norpurpureine and purpureine (thalicsimidine). Norpurpureine was found to be the major anti-platelet compound of *A. purpurea* leaves, showing activity against ADP, collagen and thrombin in human platelets. Purpureine did not inhibit human platelet activation.

Chang et al. [7] reported that 100 μg/ml (269.2 μM) norpurpureine completely inhibited the actions of arachidonic acid, collagen and platelet-activating factor (PAF) but only partially inhibited (30%) the action of thrombin. They also found that 100 μg/ml (259.4 μM) purpureine had variable inhibitory potency against arachidonic acid (85%), collagen (63%) and PAF (40%) and no effect against thrombin in rabbit platelets. Thus far, it seems that norpurpureine (at 250 μM) is a nonselective inhibitor of human and rabbit platelets with better a platelet anti-aggregating profile than purpureine. It is noteworthy that purpureine inhibits rabbit platelets with greater effect than human platelets, suggesting that significant differences may exist between rabbit and human platelets at the level of its unknown molecular target.

The anti-platelet effects of norpurpureine and purpureine analyzed in terms of structure–activity relationships indicate the lack of a methyl group at the nitrogen in norpurpureine as the key feature by which these aporphine alkaloids interact with their molecular targets. This agrees with Chia et al. [25], who found that a small change in the structure of different sub-types of isoquinoline alkaloids caused significant changes in anti-platelet aggregation activity. On the other hand, by sharing the majority of their molecular structure, these alkaloids should also share most of their non-specific interactions, which makes the anti-platelet actions of norpurpureine less likely to be mediated by the induction of non-specific interactions in membrane fluidity, as suggested for several bioactive natural products [26].

As an anti-platelet agent, norpurpureine proved pharmacologically active from 20 to 220 μM, with a potency of 80 μM, and an IC_50_ value lower than that of aspirin (140 μM) and ticlopide (510 μM) obtained under similar in vitro conditions [27]. Importantly, norpurpureine was pharmacologically effective (220 μM) in all 30 human platelet samples tested, which is evidence of its effectiveness and reveals that, at least 10 min prior to and during the 10 min of the aggregation response, it does not seem to be affected by the variability in oxidation and lipid state of these 30 PRP samples. Moreover, norpurpureine also gradually inhibited platelet granule secretion and adhesion of activated platelets to adhesive proteins like fibrinogen, suggesting that beyond hemostasis and thrombosis, this alkaloid could also modulate inflammatory and immunomodulatory activities, where these platelet functions have essential roles, particularly mediating intercellular communication [28].

Importantly, the cytotoxicity assessment of norpurpureine (100 μg/ml for 48 h) using the sulforhodamine B assay (available as Additional file 3) was promising. The compound reduced the initial cell populations of rhesus monkey kidney cell line MA104, human colon adenocarcinoma cell line HT29 and breast cancer mouse cell line 4 T1 by less than 10%. Additionally, the cytotoxicity assessment of norpurpureine (for 72 h) using alamar blue assay reports an IC_50_ value of 48.18 μM for peripheral blood mononuclear cells (PBMCs) [29]. Thus, it is likely that the anti-platelet effects of norpurpureine, exerted in 10 min, correspond to pharmacological rather than toxicological effects.

The three agonists used in this study act through different receptors and signal transduction mechanisms: ADP acts via Gαq-mediated P2Y_1_ and Gαi-mediated P2Y_12_ receptors; collagen acts mainly through tyrosine kinase-mediated immunoglobulin GP VI; and thrombin through Gα(q,12 and i_o_)-mediated PAR_1_ and Gα(q,12)-mediated PAR_4_ receptors [30]. Activation of these receptors triggers different signaling pathways that converge into common signaling events to stimulate platelet shape change, granule secretion and aggregation to support platelet function. Thus, the observation that norpurpureine inhibits the actions of three different agonists with similar potency (IC_50_ around 80 μM), strongly suggests that its molecular target should be a common downstream effector of the signaling pathways activated by these agonists.

Since norpurpureine gradually affected the amplitude of transient elevation in [Ca^2+^]_i_ induced by thrombin, its mechanism of action likely involves the negative regulation of the agonist-stimulated raise in [Ca^2+^]_i_. This correlates well with its potency to inhibit the second wave of platelet aggregation and granule secretion, and the adhesion of activated platelets to fibrinogen. In platelets, as in other non-excitable cells, increments in [Ca^2+^]_i_ involve the release of Ca^2+^ sequestered in the dense tubular system (DTS, the equivalent of the endoplasmic reticulum in platelets), followed by Ca^2+^ influx through the plasma membrane, a process referred as store-operated calcium entry (SOCE) [22]. Thus, norpurpureine actions probably involve the negative regulation of Ca^2+^ release from the DTS.

Activation of platelets by ADP and thrombin (G protein-coupled receptors) is via phospholipase C beta (PLCβ), while collagen (protein-tyrosine kinase receptor, GPVI) acts via PLCγ(2) [30]. PLC activation generates inositol 1,4,5-trisphosphate (IP_3_) and diacylglycerol (DAG) from phosphatidylinositol 4,5-bisphosphate (PIP_2_), IP_3_ activates its receptors (IP_3_-R) on the DTS to release Ca^2+^ into cytosol. DAG, together with Ca^2+^, activates PKC allowing downstream PKC-dependent events that regulate different steps during platelet activation [23]. It is interesting that the PKC activator PMA, a DAG analog, fully rescued the aggregation response inhibited by norpurpureine in platelets stimulated by ADP and collagen but only partially rescued that response in platelets stimulated by thrombin. Human platelets express at least seven of the 12 PKC isoforms, namely conventional PKCα, PKCβI, PKCβII (regulated by both DAG and Ca^2+^) and novel PKCθ, PKCη’, PKCδ and PKCε (regulated only by DAG) [31]. Thus, a specific PKC isoform (or may be upstream of PKC, at the PLC level) could be the molecular target of norpurpureine. However, additional detailed studies will be required, since the specific PKC isoforms activated downstream of each receptor are not clearly understood and PKC play isoform-specific inhibitory and stimulatory roles in platelet activation [23].

Agonist-induced reduction in cAMP is a key signaling step to remove the negative regulation of cAMP-dependent protein kinase (PKA) on calcium-related signaling elements, such as PLC-β3 [32] and IP_3_ receptors [33]. Under our experimental conditions, norpurpureine did not significantly modify intra-platelet cAMP in resting platelets, but significantly prevented the reduction in cAMP levels induced by the agonists used. Similar results were observed for IBMX, which strongly suggests the ability of norpurpureine to prevent the activation of PDEs in platelets. Human platelets express three PDE isoenzymes (PDE2, PDE3 and PDE5) and cAMP is hydrolyzed by PDE2 and PDE3 [34]. PDE3A is the most abundant isoform in platelets and has a ~ 250-fold lower Km for cAMP than PDE2 [35]. Different platelet agonists, including thrombin, significantly enhance the activity of PDE3A in a phosphorylation-dependent manner, actions that require the activation of PKC [36]. Further examination is needed to determine whether norpurpureine targets a PDE isoform to potentiate the negative regulation of cAMP on Ca^2+^ homeostasis or regulates cAMP levels via PKC.

Beyond platelets, anti-plasmodial activity [37] and in vitro cytotoxic activity toward the tumor cell lines [29] have been reported for norpurpureine. So far, no other types of biological activities have been reported for purpureine. Based on our results, in future studies it will be interesting to explore the effect of these alkaloids on the activity of different PLC, PKC and PDE isoforms in human and rabbit platelets, to have additional evidence on their structure–activity relationships and their molecular mechanisms as anti-platelet agents.

## Conclusions

We have shown for the first time that the benzylisoquinoline alkaloid norpurpureine, unlike purpureine, acts as a non-selective inhibitor of human platelet activation. The in vitro pharmacological profile of norpurpureine as anti-platelet agent is: IC_50_ value of 80 μM (potency); capacity to inhibit the action of three strong agonists of in vivo human platelet aggregation (efficacy); effective in at least 30 samples of platelets in plasma samples (PRP) from healthy donors (effectiveness). This in vitro pharmacological profile will help to support future studies of norpurpureine as an anti-thrombotic agent using animal models to establish its pharmacokinetic and pharmacodynamic profiles. Finally, we provide evidence that the molecular target of norpurpureine could be a common effector between Ca^2+^ and cAMP signaling, such as the PLC-PKC-Ca^2+^ pathway and PDEs. This needs further evaluation at the protein isoform level.

## Additional files

Additional file 1: NMR spectra norpurpureine and purpureine. (PPTX 165 kb)

Additional file 2: MS spectra norpurpureine and purpureine. (PPTX 63 kb)

Additional file 3: Cytotoxicity assessment of norpurpureine. (PDF 28 kb)

## Abbreviations

ADP: Adenosine 5′-diphosphate
AMP: Adenosine 5′-monophosphate
ATP: Adenosine 5′-triphosphate
cAMP: Cyclic adenosine 3′-5′-monophosphate
DAG: Diacylglycerol
DMSO: Dimethyl sulfoxide
DTS: Dense tubular system
EE: Ethanolic extract
Fura-2-AM: Fura-2 acetoxymethyl ester
IBMX: 3-isobutyl-1-methylxanthine
IC_50_: Half maximal (50%) inhibitory concentration
IP_3_: Inositol 1,4,5-trisphosphate
NMR: Nuclear magnetic resonance
PAF: Platelet activating factor
PDEs: Phosphodiesterases
PIP_2_: Phosphatidylinositol 4,5-bisphosphate
PKA: cAMP-dependent protein kinase
PKC: Protein kinase C
PLC: Phospholipase C
PMA: Phorbol 12-myristate 13-acetate
PRP: Platelet-rich plasma
