# Supplementary material for: The norpurpureine alkaloid from Annona purpurea inhibits human platelet activation in vitro
Source: Cell Mol Biol Lett. 2018 Apr 18;23:15. doi: 10.1186/s11658-018-0082-4 (PMC5905151; doi:10.1186/s11658-018-0082-4)
Supplement: Supplementary file 1 — NMR spectra norpurpureine and purpureine. (PPTX 165 kb) [file 11658_2018_82_MOESM1_ESM.pptx]

## Slide 1
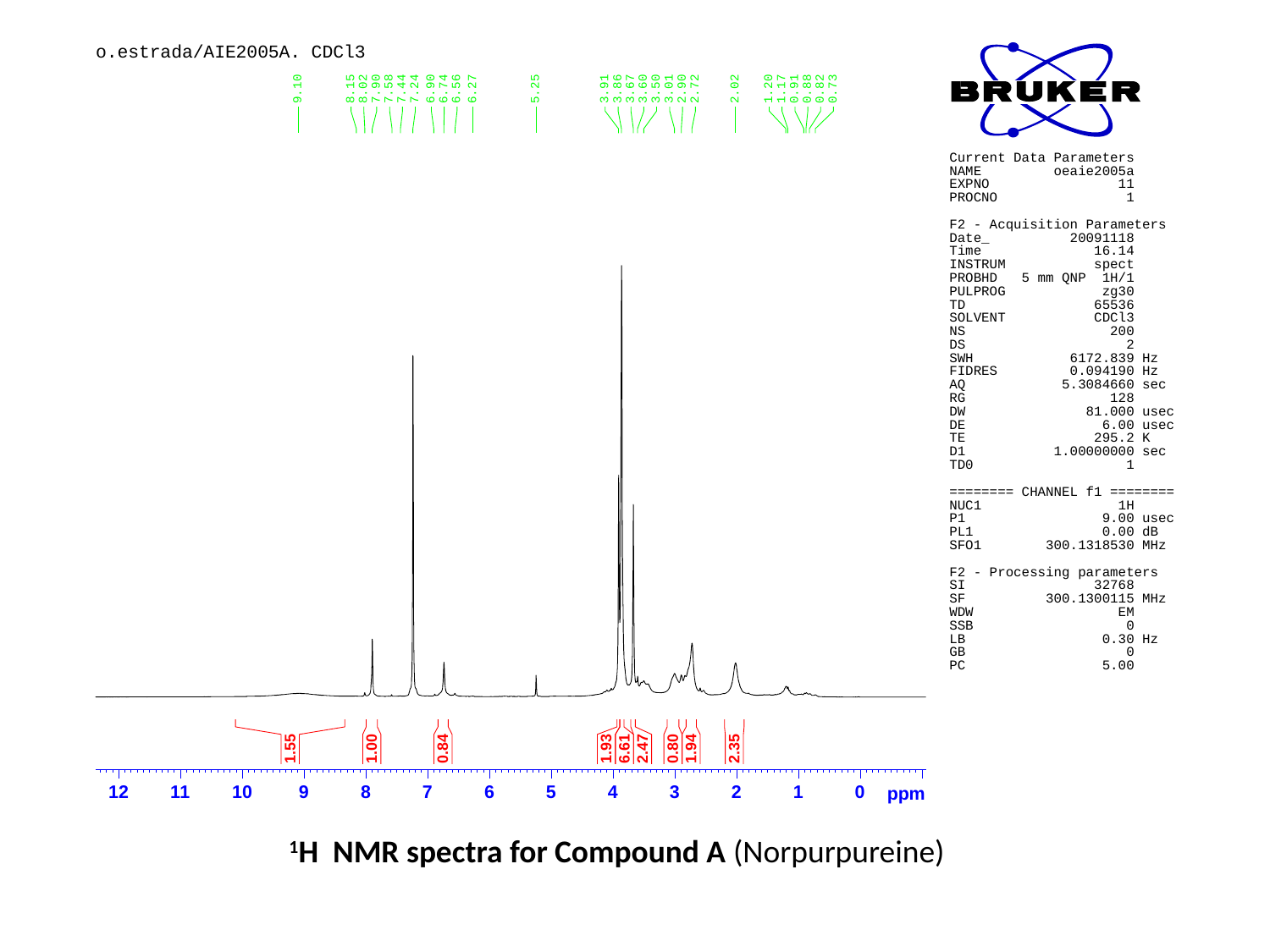

1H NMR spectra for Compound A (Norpurpureine)

## Slide 2
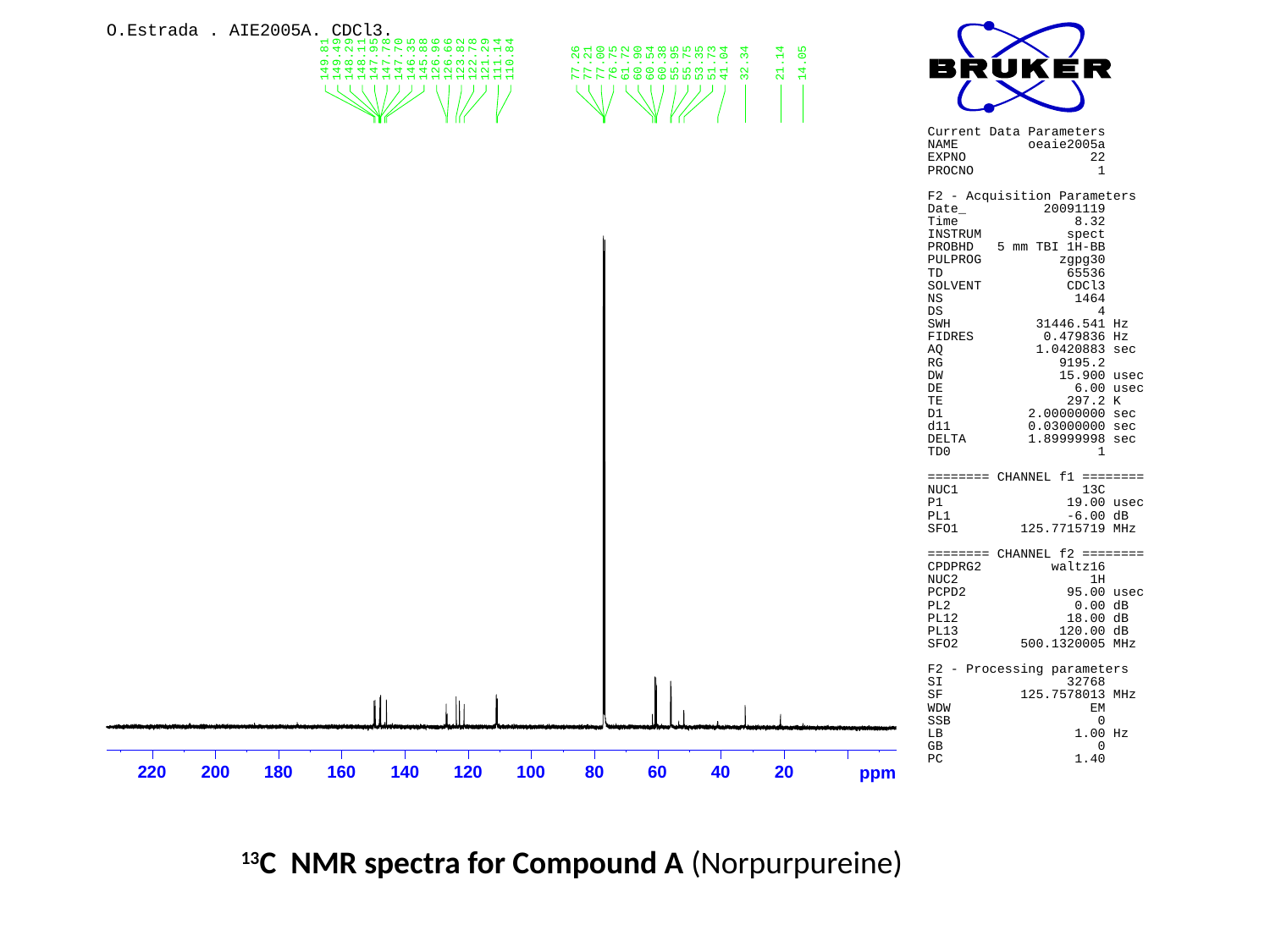

13C NMR spectra for Compound A (Norpurpureine)

## Slide 3
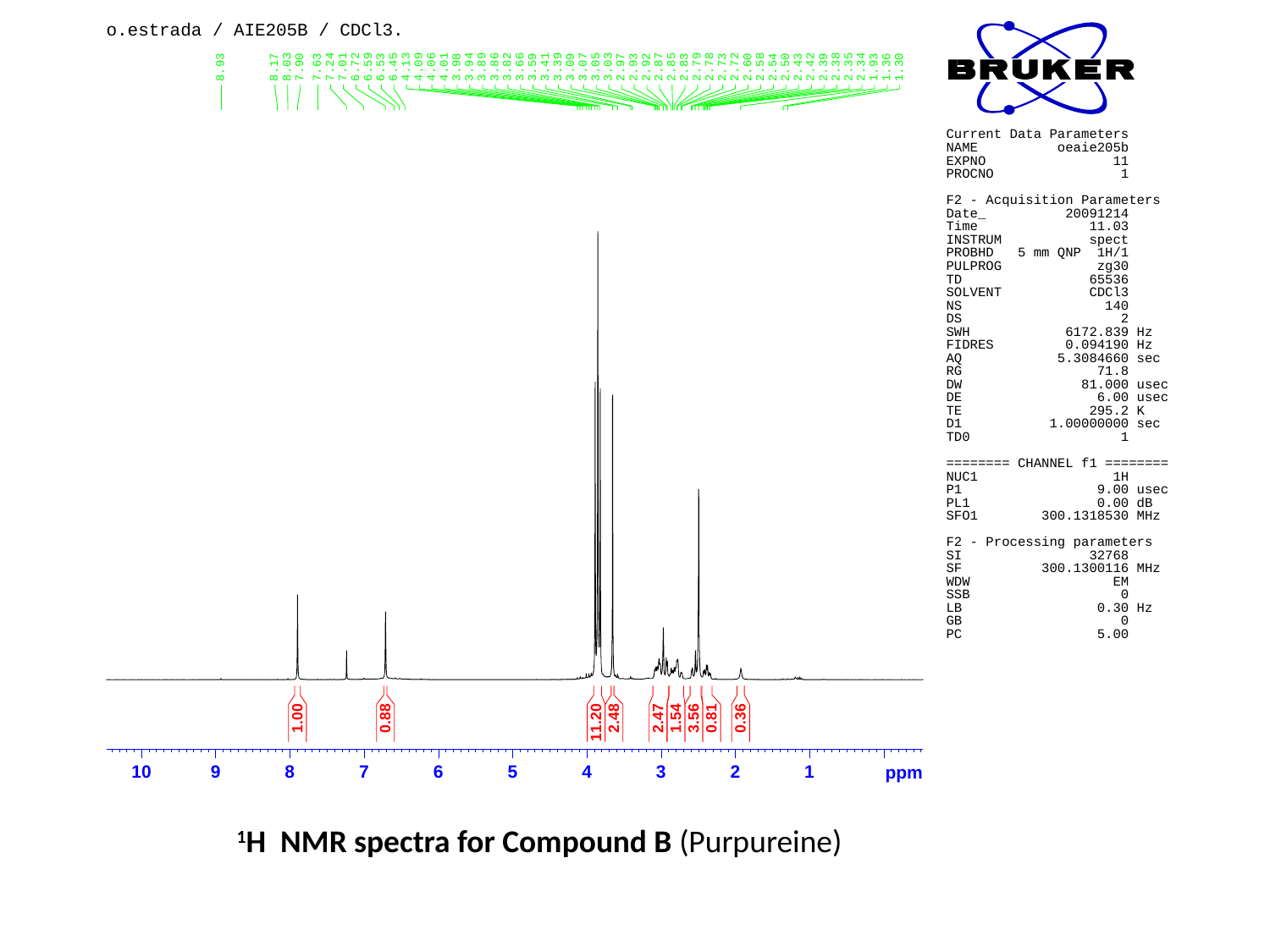

1H NMR spectra for Compound B (Purpureine)

## Slide 4
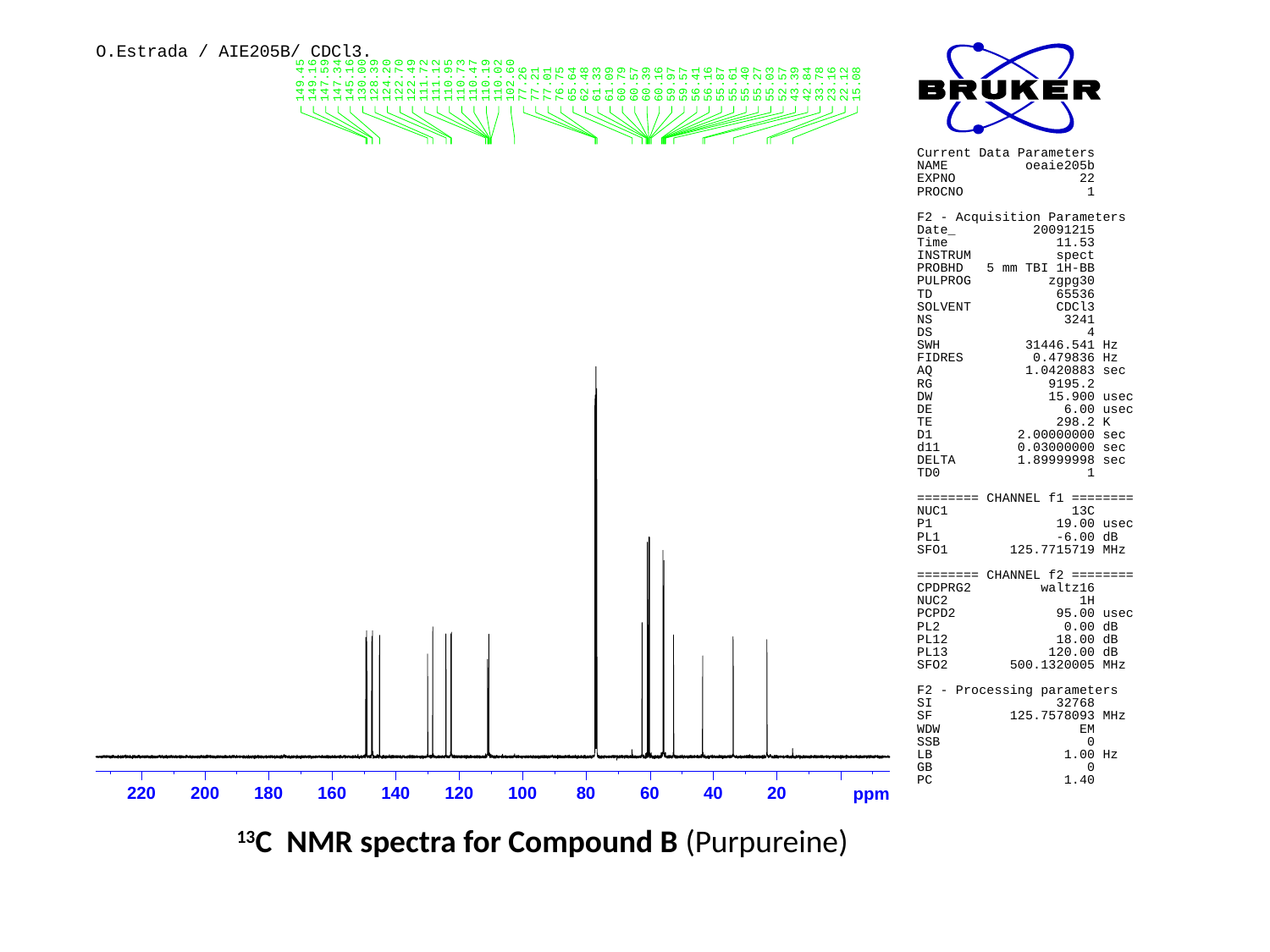

13C NMR spectra for Compound B (Purpureine)
