# Supplementary figures and images for: The norpurpureine alkaloid from Annona purpurea inhibits human platelet activation in vitro
Source: Cell Mol Biol Lett. 2018 Apr 18;23:15. doi: 10.1186/s11658-018-0082-4 (PMC5905151; doi:10.1186/s11658-018-0082-4)

## Slide 1
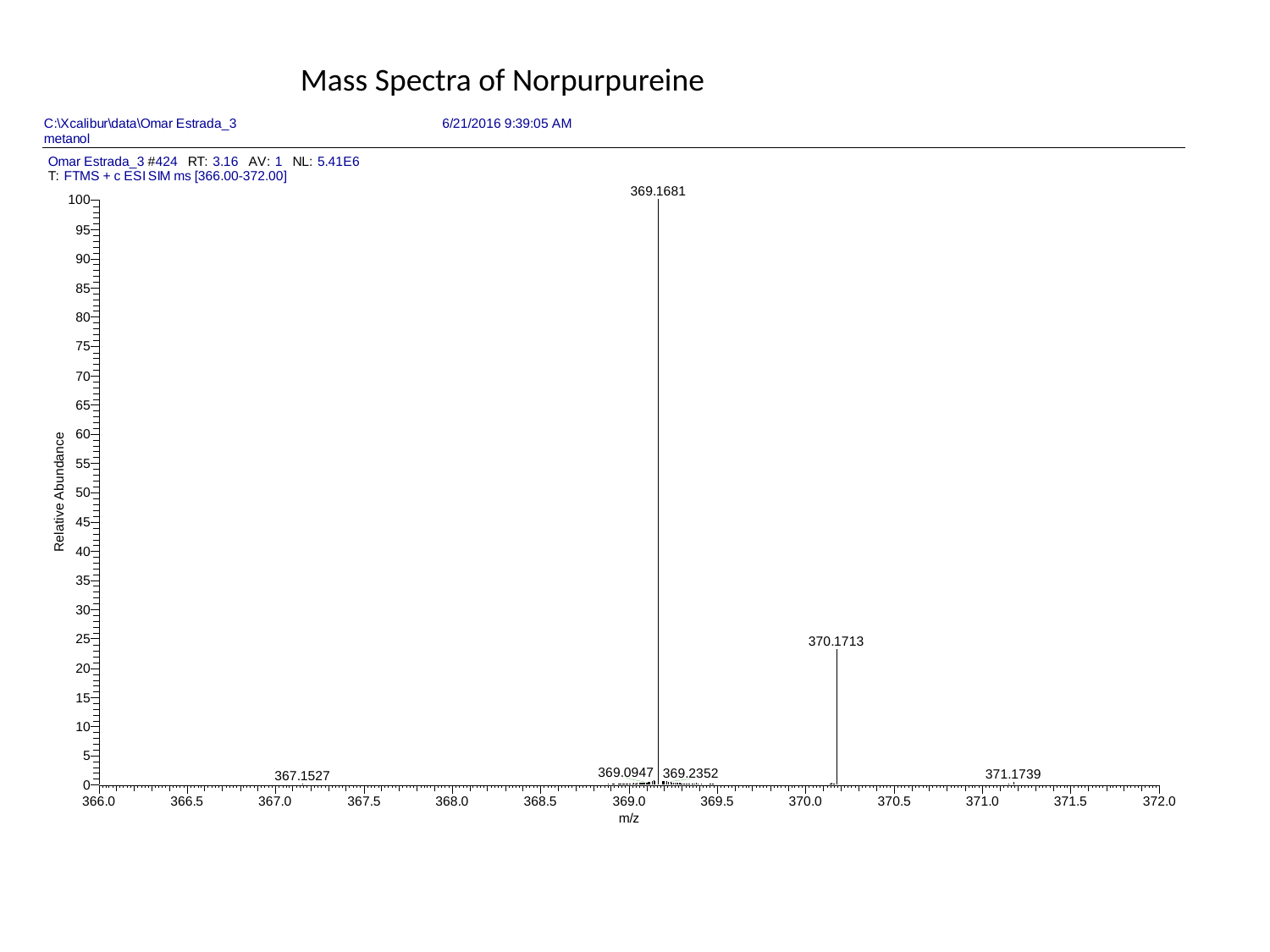

Mass Spectra of Norpurpureine

## Slide 2
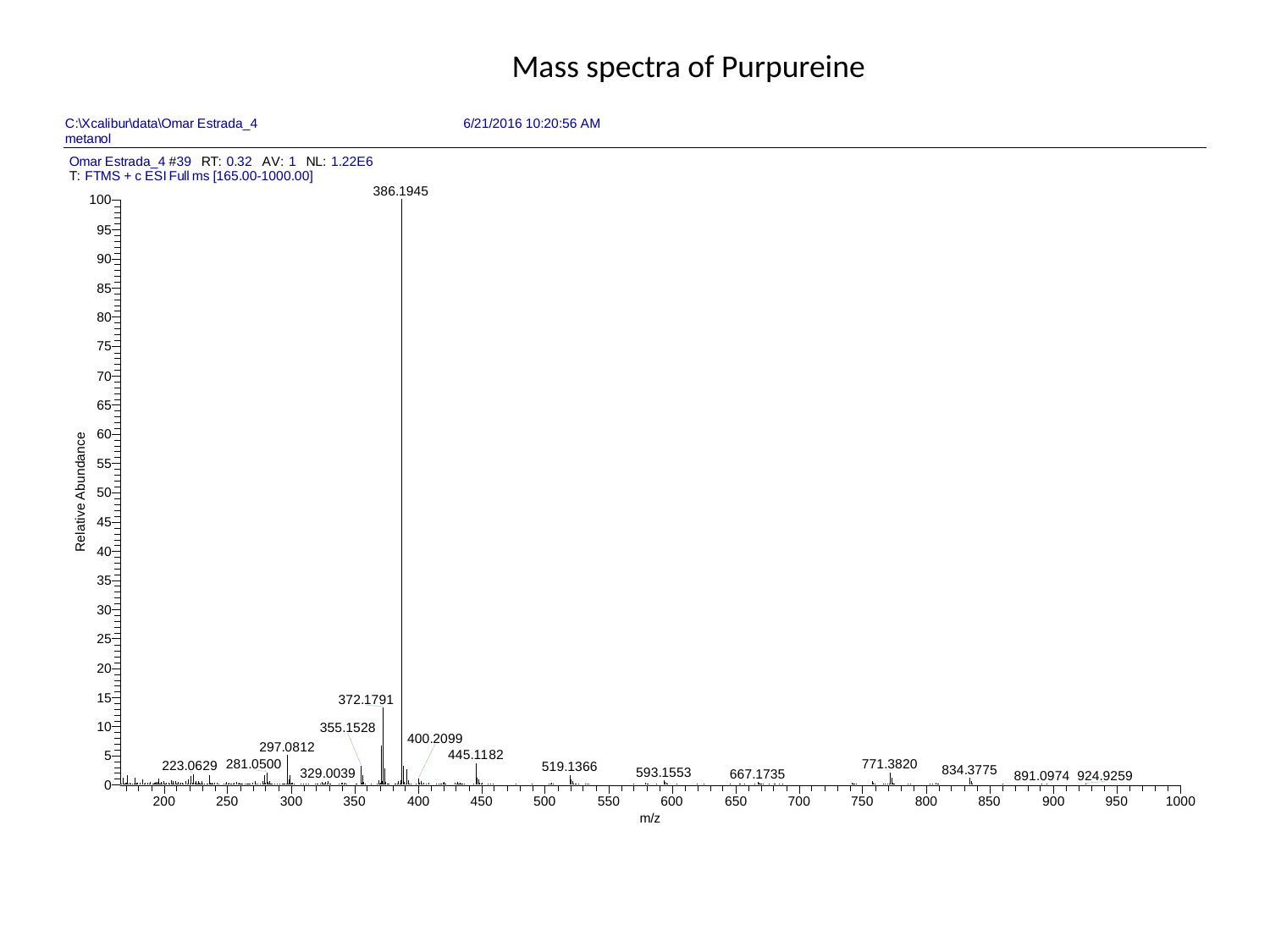

Mass spectra of Purpureine

Supplement: Supplementary file 2 — MS spectra norpurpureine and purpureine. (PPTX 63 kb) [file 11658_2018_82_MOESM2_ESM.pptx]
