# Supplementary material for: The norpurpureine alkaloid from Annona purpurea inhibits human platelet activation in vitro
Source: Cell Mol Biol Lett. 2018 Apr 18;23:15. doi: 10.1186/s11658-018-0082-4 (PMC5905151; doi:10.1186/s11658-018-0082-4)
Supplement: Supplementary file 3 — Cytotoxicity assessment of norpurpureine. (PDF 28 kb) [file 11658_2018_82_MOESM3_ESM.pdf]

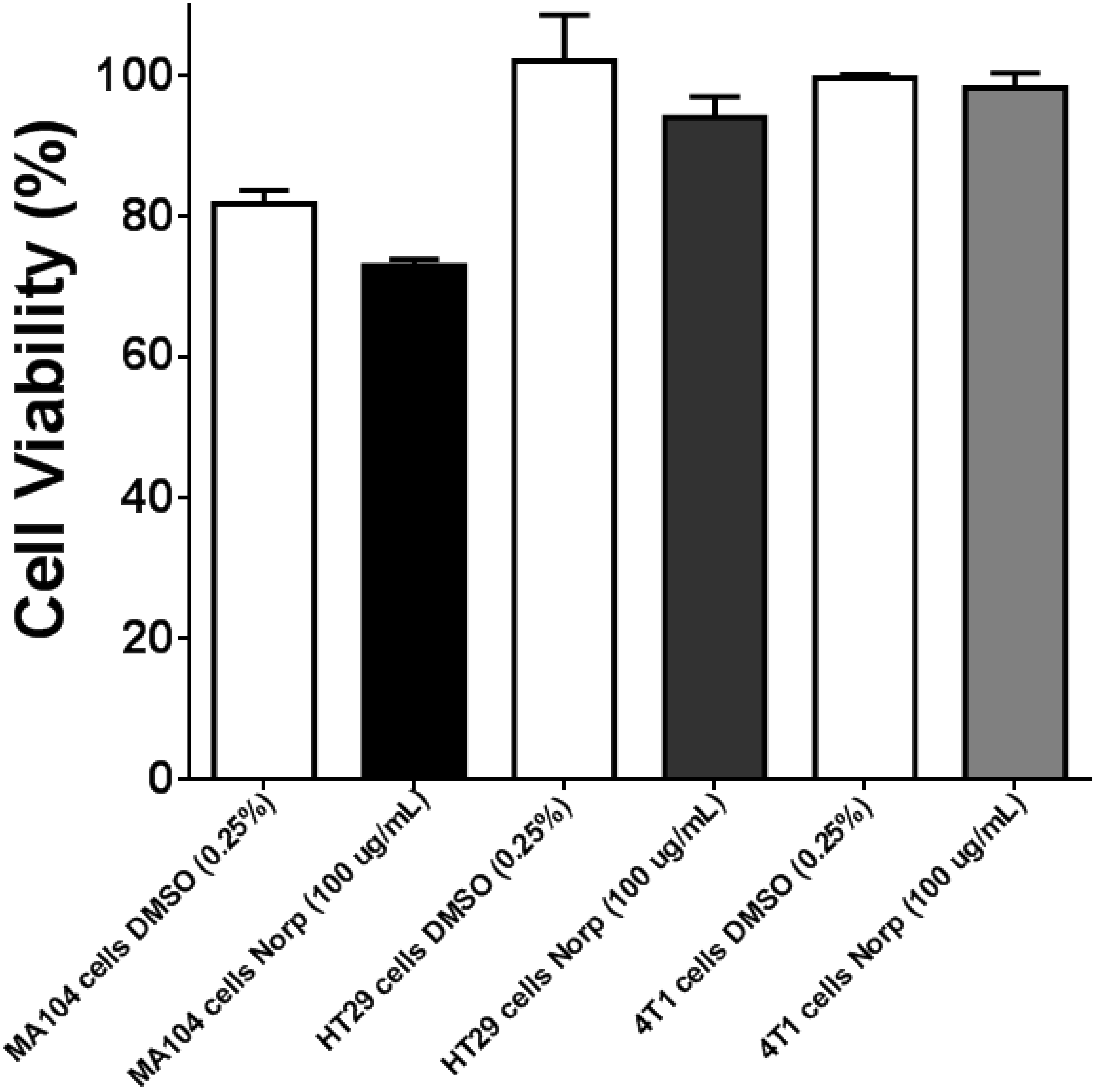

Effect of norpurpureine on cell viability in three cell tumor lines. Cells were pretreated with DMSO (0.25%) or 100 μg/ml of norpurpureine for 48 hours before the total protein content were evaluated by sulforodamine B. The data are the mean (SD) (n = 3, done in triplicate).
